# Cone beam computed tomography based upper airway measurement after orthognathic surgery: a comparative evaluation of different imaging software

**DOI:** 10.1038/s41598-024-83890-7

**Published:** 2025-02-24

**Authors:** Andreas Helmuth Iti Mini, Hannes Wegner, Daniel Lonic, Denys J. Loeffelbein

**Affiliations:** 1 Polyclinic for Maxillofacial Surgery, Technical University of Munich, University Clinic Rechts der Isar, Ismaningerstr. 22, 81675 Munich, Germany; 2 MCLINIC, Interdisciplinary Specialist Center, Am Schützeneck 8, 81241 Munich, Germany; 3 Clinic for Maxillofacial Surgery and Plastic Surgery, Helios Clinic Munich West, Steinerweg 5, 81241 Munich, Germany; 4 Department of Plastic, Hand, and Reconstructive Surgery, University of Regensburg, Regensburg University Hospital, Regensburg, Germany

**Keywords:** Orthognathic surgery, Cone-beam computed tomography, Upper airway, Obstructive sleep apnea syndrome, Software comparison, Signs and symptoms, Oral manifestations, Respiratory signs and symptoms, Anatomy, Oral anatomy, Medical research, Outcomes research

## Abstract

Cone-beam computed tomography (CBCT) enhances understanding of the upper airway (UA). This study compared three software products’ abilities in visualizing and quantifying specific upper airway changes using CBCT. We conducted a retrospective analysis of pre- and post-operative CBCT images from 29 patients using Dolphin (Do), Romexis 5 (Ro5), and Romexis 6 (Ro6) software, focusing on alterations in oropharyngeal volume and minimum cross-sectional area as key indicators of orthognathic surgery outcomes. ANOVA analysis showed significant differences in volume measurements between Do/Ro5 (*p* = 0.034) and Do/Ro6 (*p* = 0.047), but no difference between Ro5 and Ro6 (*p* = 0.685). No significant differences were found in minimum cross-sectional area parameters. Despite standardized protocols, interpretation discrepancies exist between Do and Ro 5/6, possibly due to program-specific properties. Further studies on threshold value comparability are needed for data standardization. Direct comparisons of clinical data from Do, Ro5, and Ro6 are limited due to methodological disparities. Nonetheless, these programs allow reproducible and quantifiable measurements for clinical assessments of these specific airway changes following orthognathic surgery.

## Introduction

Obstructive sleep apnea syndrome (OSAS) is a prevalent condition affecting approximately 936 million people worldwide, with an increasing trend^1^. While obesity is recognized as the primary risk factor^2^, particularly due to increased cervical circumference which, combined with sleep-related hypotonia of pharyngeal muscles, can lead to upper airway collapse, other contributing factors include narrow pharyngeal structures, reduced upper airway (UA) volumes, and impaired pharyngeal muscle function^3^. Jaw misalignments and genetically induced jaw anomalies can also lead to dysfunctional airways^4^. Untreated OSAS is associated with numerous long-term health consequences^5–10^.

Accurate diagnosis and assessment of OSAS and related UA abnormalities are crucial for effective treatment planning. While polysomnography remains the gold standard for OSAS diagnosis, radiographic imaging techniques serve as valuable diagnostic complements. Cone beam computed tomography (CBCT) has emerged as a widely utilized method for visualizing the UA due to its lower radiation dosage compared to conventional computed tomography (CT). Furthermore, CBCT enables the calculation of volumes and cross-sectional areas, providing more comprehensive data than the linear measurements obtained from two-dimensional cephalometry^11,12^.

The advancement of CBCT image viewing software, coupled with the integration of surface scanning techniques and open file formats such as STL, has established CBCT as a secure planning basis for virtual surgical simulations in maxillofacial corrections^13^. This is particularly relevant in the context of orthognathic surgery, which has shown promising results in treating OSAS. Maxillomandibular advancement (MMA), for instance, has demonstrated a success rate of approximately 86% and a cure rate of about 40% in adult OSAS patients^14–16^.

Given the invasive nature of jaw repositioning procedures and their potential side effects, it is crucial for both patients and clinicians to employ precise methods to understand post-operative changes in the UA. CBCT scans allow for quantification of these changes through volume and cross-sectional area measurements. However, it remains unclear whether available software programs interpret these effects comparably.

Despite the widespread use of CBCT for UA visualization in orthognathic surgery planning, a significant research gap exists regarding the comparability of measurements across different software programs. This study aims to address this gap by comparing three widely used programs: Dolphin (Imaging & Management Solutions, Chatsworth, USA), Romexis 5, and Romexis 6 (both Planmeca Oy, Helsinki, Finland). Our primary objective is to quantify potential differences in the interpretation of changes in oropharyngeal volume and minimum cross-sectional area following orthognathic interventions, thus contributing to the standardization of CBCT-based airway analyses.

## Materials and methods

The present study is a retrospective investigation based on CBCT scans obtained during the period from 01.01.2017 to 16.06.2022 as part of maxillofacial surgical therapies. Ethical approval was waived by the local Ethics Committee of Technical University of Munich in view of the retrospective nature of the study, and all the procedures performed were part of routine care (14.06.2022/2022-251-S-NP). According to the local Ethics Committee of Technical University of Munich, no informed consent was needed for the use of the CBCT scans of all patients. All methods were performed in accordance with the WMA declaration of Helsinki. The scans comprise preoperative (t0) and postoperative (t1) images, which are utilized for treatment planning, simulation, and assessing treatment outcomes. The postoperative Image was taken a minimum of 3 months and a maximum of 12 Months after the surgery. The sample size for this study was determined through an a priori power analysis. Assuming a large effect size (Cohen’s d = 0.8) for the differences in airway measurements between software programs, with a desired power of 0.80 and a significance level of α = 0.05, the analysis was conducted. The study included participants aged 18–65 years who had not undergone previous orthognathic surgery and for whom high-quality CBCT scans were available. Participants with severe skeletal deformities were excluded from the study. Additionally, CBCT scans showing significant artifacts or poor image quality were not considered for analysis. A total of 29 patients who met the inclusion criteria were enrolled in the study, including 15 males and 14 females. These patients underwent five different surgical therapies. 6 maxillary advancements, 11 mandibular advancements, 5 maxillo-mandibular advancements, 5 maxillary advancements with mandibular retraction and 2 mandibular retractions.

The UA were assessed through measurements of volume (V) and minimal cross-sectional area (MCA), focusing on the oropharynx region. Three distinct measurement methods employing three different software packages were utilized: Dolphin (Do), Romexis 5 (Ro5), and Romexis 6 (Ro6). Volume and MCA were measured for each patient using each software at two time points: T0 (preoperative) and T1 (postoperative). Each of these measurements was performed twice (M1 and M2) by the same assessor. A minimum interval of 24 h was maintained between the first and second measurements, which was deemed sufficient to mitigate recall bias. No predefined interval was set for all measurements beyond this minimum, as this approach more closely reflects real clinical scenarios. The results of the first and second measurements were recorded in separate Excel spreadsheets and only combined after all patient cases had been fully processed. To determine the change in variables following surgery, differences were calculated between T0 and T1 (Diff.T1-T0). This comprehensive approach ensures a thorough evaluation of the UA changes while maintaining a protocol that closely mimics clinical practice.

### Cone beam computed tomography

All images were part of the therapy and were not specifically acquired for this study. All CBCT scans utilized were obtained at MCLINIC (Munich) using the ProMax® 3D Mid + Pan device (Planmeca Oy, Helsinki, Finland) (120 kV; 3.6 mA; 993 Gy·cm^2^; 9.151 s.; 200 × 170 mm FOV). All CBCT scans were conducted with patients standing, with their head fixed, and positioned according to the Frankfurt Horizontal plane^17^. Patients were instructed to place their tongues on the palate, remain still, and breathe gently through the nose during the scanning process. The voxel size was 400 µm. The technical parameters of the standardized measurement protocol were the following: Anatomic region of interest: Oropharynx. Image alignment was standardized across all three planes to ensure consistency in measurements. In the sagittal plane, the image was oriented so that the line connecting the anterior nasal spine (ANS) and posterior nasal spine (PNS) was horizontal. For the axial plane, alignment was achieved by ensuring that the horizontal line connecting the most inferior points of both orbits was parallel to the image frame. In the coronal plane, the image was oriented so that the line connecting the ANS and PNS appeared vertical. This standardized alignment procedure was applied consistently across all measurements and software programs, providing a uniform basis for comparison. The region of interest (ROI) for the oropharyngeal airway volume (V) was defined as follows: The coronal boundary was established by an imaginary plane connecting the anterior nasal spine (ANS) and the posterior nasal spine (PNS). This plane served as the superior limit of the measured volume. The caudal boundary was defined by a tangential plane touching the most inferior point of the third cervical vertebra (C3). This plane represented the inferior limit of the measured volume. The anterior and posterior boundaries were determined by the soft tissue interfaces of the pharyngeal airway. Within this defined three-dimensional space, the minimal cross-sectional area (MCA) was identified as the smallest axial cross-section of the airway. These standardized anatomical landmarks were used consistently across all software programs to ensure comparability of measurements.

### Definition of anatomical segments

The UA comprise the nasal cavity, nasopharynx, oropharynx, and hypopharynx. Several studies have previously highlighted the absence of a universally accepted anatomical definition of the airways^18–21^, resulting in inconsistencies in the literature regarding the delineation of the UA. Most authors define the oropharynx as the region between the soft palate and the upper edge of the epiglottis^18^. In contrast, others have designated the hyoid bone as the lower boundary of the oropharynx^22^. For the present study, the definition provided by the Prometheus Atlas was adopted^23^.

### Landmarks and image alignment

The boundaries of the region of interest (ROI) must be determined via CBCT based on anatomical landmarks. For precise and reproducible procedures, landmarks on bony structures are preferable^24^. Even better if they are sharply defined from surrounding tissue with high contrast and taper in shape^25^. For comparison of measurements at different time points, it is essential, due to varying patient positions during repeated scans, to align the images according to a consistent principle for each measurement. To this end, the imaginary line connecting the bony landmarks of the nasalis anterior and posterior spina was aligned parallel to the horizontal coordinate axis. This method aligns with the findings of existing reviews on airway measurements in CBCT scans^19^.

### Dolphin

**Fig. 1:**
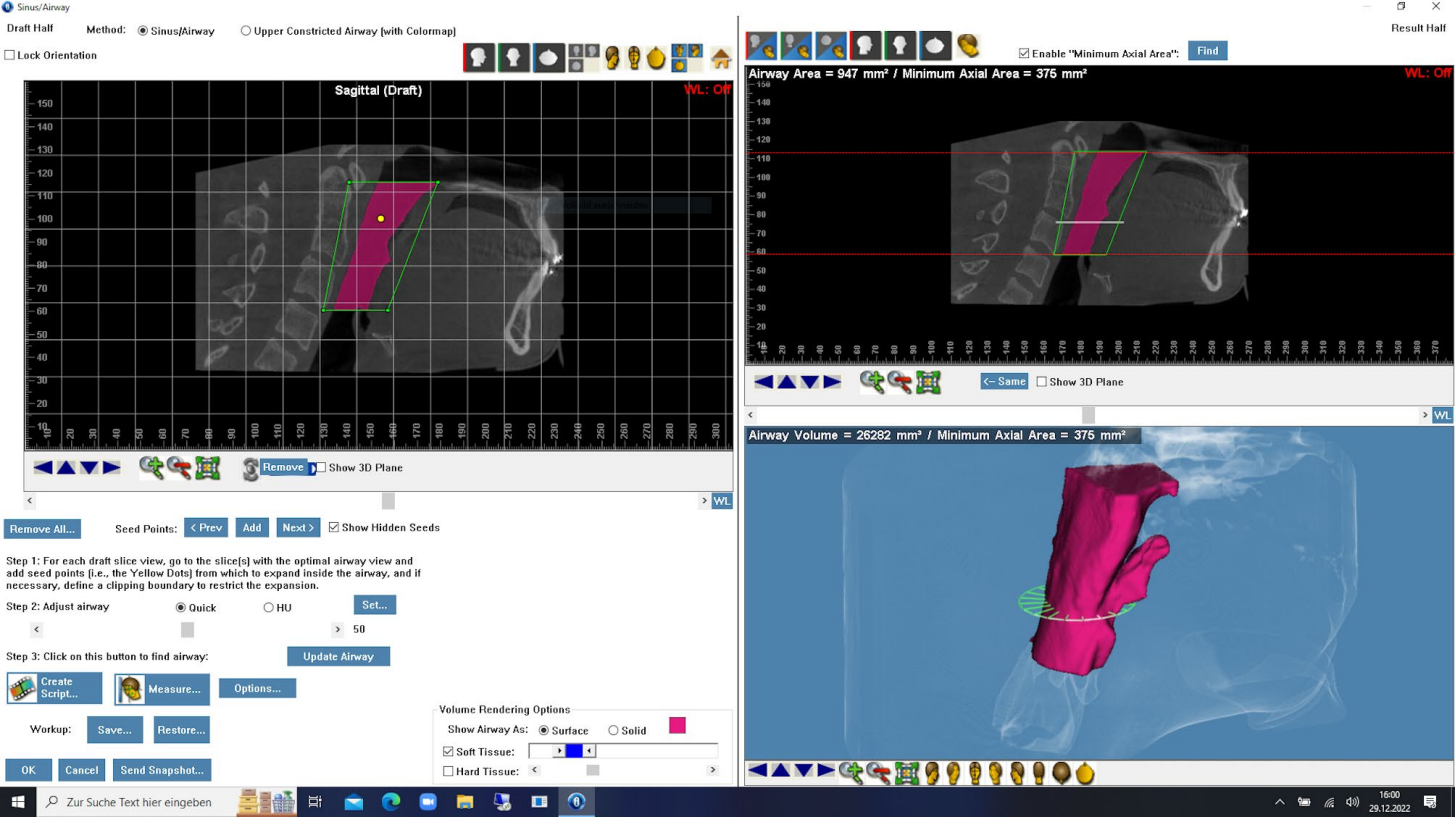
Dolphin Airway Measurement Tool; Screenshot. Dolphin Imaging software is designed for use by specialized dental practices for capturing, storing and presenting patient images and assisting in treatment planning and case diagnosis. Shown is a screenshot of one of the modules within the software, the airway measurement tool. In this case the measurement was already accomplished. ROI is shown in the upper left window. Measured volume is displayed in violet, including a three-dimensional model of measured UA in the lower right window. Minimal cross-sectional area is shown in the upper right window.

Dolphin software provides a specialized tool for airway analysis within which the image is aligned according to the protocol. The ROI was defined by a freely constructible polygon within the image. The measurement area was determined by selecting seed points. The gray value threshold was set at 65 for this study. Dolphin presents the result in the form of a 3D model, including the value for V. The determination of the MCA was made in a separate sagittal view of the model (see Fig. 1).

### Romexis 5

The image was aligned according to the measurement protocol. The Regional Growth Tool (RGT) was utilized for the measurements. The ROI was defined by delineating a cube in the sagittal view. The threshold was set to the program’s default for “air” (300). In the views of the three planes, the measured airway is shaded in color according to its cross-sectional area. The MCA needs to be manually determined by scrolling through the horizontal coordinate within the red area and reading the value. V is automatically provided in an information box.

### Romexis 6

**Fig. 2:**
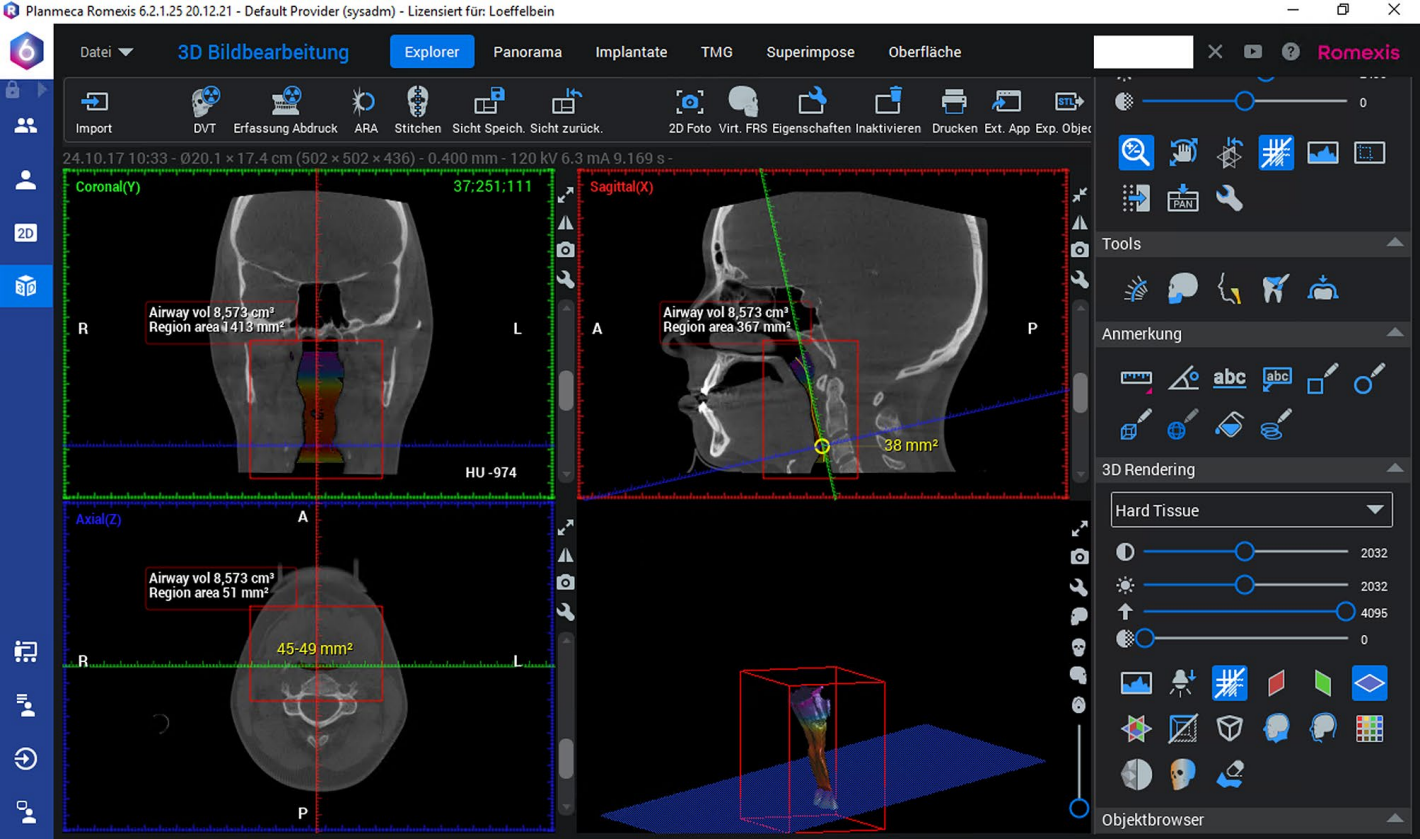
Romexis 6 Airway Extraction Tool; Screenshot. Romexis 6 software is designed for use by specialized dental practices for capturing, storing and presenting patient images and assisting in treatment planning and case diagnosis. Shown is a screenshot of one of the modules within the software, the airway extraction tool. In this case the measurement was already accomplished. Upper left window shows axial view, lower left window the horizontal view. Upper right window shows sagittal view with measurement values in a box and cross lines according to axis of MCA measurement. Lower right window shows three-dimensional model of measured UA.

In this method, the Airway Extraction Tool (AET) was used. The image was aligned according to the specifications of the measurement protocol. To define the ROI, it is necessary to mark a chain of points freely along the course of the airway. The points are linked automatically to form the measurement line (ML). The first and last points define the cranial and caudal boundaries of the ROI. Subsequently, the ROI was automatically defined by the program. The threshold was set to the default program setting for “air” (500). The calculation of V and the MCA is performed automatically (see Fig. 2).

### Statistical evaluation

The results of the measurements were collected for each program and measurement series using Excel in separate tables and then transferred to and analyzed in SPSS. Normal distribution was tested with Shapiro–Wilk test. The methods were compared in Bland‒Altman plots (BAPs). For a statistical comparison of the data, one-way analysis of variance (ANOVA) was performed. The intrarater reliability was assessed by calculating the intraclass correlation coefficient (ICC), which was determined using a model with mixed two-way effects. The model employs a definition of absolute agreement.

## Results

The changes in oropharyngeal variables V and MCA following orthognathic surgery were interpreted differently by the tested programs. The Dolphin program interprets the change in V to be greater than that of the two Planmeca programs. In contrast, Romexis 6 interpreted the change in MCA to be greater than that of Romexis 5 and Dolphin (Tables 1 and 2).

**Table 1:** Comparison of volumetric measurements of oropharyngeal airways among different software programs.

Software	N	Mean	SD	95% CI	Dolphin	Romexis 5	Romexis 6
Dolphin	29	4.094	1.379	1.270–6.918	–	0.034*	0.047*
Romexis 5	29	2.697	1.227	0.182–5.211	0.034*	–	0.685
Romexis 6	29	2.912	1.214	0.424–5.399	0.047*	0.685	–

*Statistically significant (*p* < 0.05).

**Table 2:** Comparison of minimal cross-sectional area measurements of oropharyngeal airways among different software programs.

Software	N	Mean	SD	95% CI	Dolphin	Romexis 5	Romexis 6
Dolphin	29	51.448	19.865	10.757–92.139	–	0.945	0.233
Romexis 5	29	52.207	17.301	16.768–87.646	0.945	–	0.069
Romexis 6	29	64.655	18.892	25.957–103.354	0.233	0.069	–

*Statistically significant (p < 0.05).

### Comparison of airway measurements across software programs

The statistical analysis of variance (ANOVA; α = 0.05) revealed significant differences for variable V between Dolphin and both Romexis programs [Do/Ro 5 (*p* = 0.034) and Do/Ro 6 (*p* = 0.047)]. There was no significant difference between the two Romexis programs [Ro 5/Ro 6 (*p* = 0.685)]. Further paired t tests (α = 0.05) for Do/Ro5 (*p* = 0.017) and Do/Ro6 (*p* = 0.024) confirmed the differences. For the MCA, there were no significant differences among the three programs (Do/Ro5: *p* = 0.945; Do/Ro6: *p* = 0.233; Ro5/Ro6: *p* = 0.069).

### Agreement and bias between software measurements

The mean bias of the V (cm^3^) and MCA (mm^2^) and relative limits of agreement (LoA) were obtained from Bland‒Altman analysis for each software comparison (Table 3) (Figures available in supplementary information).

**Table 3:** Bland altman analysis V = cm^3^, MCA = mm^2^.

	(V) Diff t1–t0 Dolphin/ Romexis 5	(V) Diff t1–t0 Dolphin/ Romexis 6	(V) Diff t1–t0 Romexis 5/Romexis 6	(MCA) Diff t1–t0 Dolphin/ Romexis 5	(MCA) Diff t1–t0 Dolphin/ Romexis 6	(MCA) Diff t1–t0 Romexis 5/Romexis 6
Mean Diff. (Midline)	1.379	1.182	− 0.215	− 0.759	− 13.207	− 12.448
Standard deviation	3.379	3.064	2.821	58.582	58.350	35.427
LoA (Upper line)	8.021	7.187	5.315	114.062	101.160	56.989
LoA (Lower line)	− 5.227	− 4.823	− 5.744	− 115.580	− 127.574	− 81.886

### Reliability and consistency of airway measurements

To assess the reliability of the methods, the measurement repetitions M1 and M2 were compared for each variable and time point. All three programs exhibited minimal differences between M1 and M2. Calculation of the Intra Class Correlation Coefficient (ICC) yielded excellent values for both variables and time points across all three software packages (Table 4). The ICC values were interpreted using the criteria proposed by Koo and Li (2016): ICC < 0.50 indicates poor reliability, 0.50 ≤ ICC < 0.75 moderate reliability, 0.75 ≤ ICC < 0.90 good reliability, and ICC ≥ 0.90 excellent reliability^26^. Individual measurements were plotted against each other in the BAP test, showing very narrow limits of agreement (Figures and tables available in supplementary information).

**Table 4:** Intraoperator reliability V = cm^3^, MCA = mm^2^.

	Dolphin				Romexis 5				Romexis 6			
	V		MCA		V		MCA		V		MCA	
	T0	T1	T0	T1	T0	T1	T0	T1	T0	T1	T0	T1
Mean	18.50	22.59	197.93	249.38	16.52	19.21	168.86	221.07	17.10	20.02	162.45	227.10
SD	6.85	7.45	113.92	113.09	6.83	6.80	105.25	100.86	6.75	6.76	99.17	106.70
ICC	0.99	0.99	0.99	0.95	0.99	0.99	0.99	0.99	0.99	0.99	0.98	0.97

## Discussion

The present study is a retrospective investigation comparing various digital methods for analyzing changes in the UA via CBCT following orthognathic surgery. The aim was to utilize software packages that are widely used in both the USA and Europe, particularly in Germany. To the best of the authors’ knowledge, no previous studies have compared the presented methods. The study design was oriented toward feasibility within clinical practice. Measurements M1 and M2 were conducted for each measurement method on different days and after training for the respective programs, solely by the same individual.

The decision to compare Romexis 5 and 6 in this study is grounded in several important considerations regarding the evolution and application of imaging software in clinical practice. Commercial imaging programs are subject to continuous development, with clinicians often working with multiple versions of the same program throughout their careers. Given the lack of available information on potential algorithmic differences between these tools, comparing their measurement accuracy and consistency is of paramount importance. This analysis is particularly relevant for clinicians and researchers who wish to compare data from different software versions or evaluate the impact of software transitions on their measurements. Furthermore, this comparison contributes to assessing methodological consistency in longitudinal studies and retrospective analyses, which is crucial for the reliability and comparability of research outcomes in the field of airway analysis using CBCT. By examining these two versions, we provide valuable insights into the potential variability in airway measurements across different iterations of the same software platform, addressing a critical aspect of both clinical practice and research continuity in the rapidly evolving landscape of dental imaging technology.

Changes in the UA following orthognathic procedures have been extensively studied using linear distances, angles, and areas^27–29^. However, a much deeper understanding of the airways and their alterations posttherapy can be achieved through three-dimensional imaging^30^. With the increasing prevalence of OSAS, investigations into the airways have increasingly adopted a three-dimensional approach, focusing on volumes and cross-sectional areas^18,31–33^. Research on three-dimensional studies in the context of obstructive sleep apnea is extensive^34–38^. Similarly, alterations in the airways following orthognathic surgery have also been thoroughly examined in three dimensions^21,39–42^.

In light of these advancements in three-dimensional imaging and analysis, it is crucial to consider the underlying algorithms used by different software versions for airway volume calculation. While the exact algorithms used by Romexis 5 and 6 for airway volume calculation are not publicly disclosed due to proprietary reasons, it is important to consider the potential differences in their approaches. The "Region Growing Tool" in Romexis 5 likely employs a seed-based region growing algorithm, which expands from a user-defined starting point to include connected voxels within a specified density range. This method allows for versatility in measuring various air-filled spaces but may require more user input and be susceptible to leakage into adjacent structures. In contrast, the "Airway Extraction Tool" in Romexis 6, being specifically designed for airways, might incorporate more sophisticated segmentation techniques, possibly including anatomical models or predefined airway templates to guide the segmentation process. These potential differences in algorithmic approaches could contribute to variations in volume measurements between the two versions. It is worth noting that the field of airway analysis is rapidly evolving, with artificial intelligence (AI) playing an increasingly significant role. Recent studies have explored the use of deep learning algorithms for automated airway segmentation and the diagnosis of obstructive sleep apnea based on both airway and facial features extracted from CBCT images^43,44^. While not directly employed in our study, these AI-driven approaches show promise in enhancing the accuracy and efficiency of airway analysis and OSAS diagnosis. Future research comparing traditional segmentation methods with AI-based techniques could provide valuable insights into the most effective tools for clinical practice and research in this field.

All the programs demonstrated excellent agreement in repeated measurements. The utilized methods allow for reproducible volume and cross-sectional measurements. The high intrarater correlation coefficient supports this finding and is consistent with the literature^45,46^.

Despite the high ICCs, this study revealed differences between the measured variables. The results for changes in the MCA following orthognathic procedures show substantial differences between Ro6 (64,66 mm^2^) and Do (51,45mm^2^) and between Ro6 (64,66mm^2^) and Ro5 (52,21 mm2), whereas Do and Ro5 show similar results. Although these differences are not statistically significant, they indicate methodological discrepancies. The determined mean values for changes in V, on the other hand, differ significantly. The mean values for Δt1–t0 for Dolphin (4,094 cm^3^) are 1.5 times and 1.4 times larger than those for Romexis 5 (2,697 cm^3^) and Romexis 6 (2,912 cm^3^), respectively, while the two Romexis versions show minimal deviation from each other. Bland‒Altman analysis revealed that Dolphin generally interpreted the change in V to be greater, which is consistent with the findings of other studies^47^. A similar trend can be observed with the Romexis 6 measuring MCA (Table 3) (supplementary information Figs. 1–6).

CBCT devices vary in terms of radiation production, filtration, and detection. The hardware significantly influences image quality. The data reconstruction method, where an algorithm averages the grayscale values for voxels, also plays a role. The grayscale values depend on the homogeneity of the structures. Manufacturer-specific modifications to the algorithm often remain unclear. Chen et al. identified significant differences in oropharyngeal measurements among different CBCT devices in 2018^48^. The present study employed uniform imaging settings to enhance comparability. To date, there is no standard protocol for creating a CBCT for airway volumetrics. It is known that head position and breathing alter the dimensions of the UA^49,50^. Patient positioning also affects airway volume^51^. In the present study, the head was aligned according to the Frankfurt Horizontal plane. All scans were conducted with the patients standing. The parameters conform to a methodology recommended for 3D airway volumetric measurements with CBCT^19^. With a scan time of only approximately 9s, motion artifacts could be minimized, still capturing multiple breathing cycles.

Volumetric measurements of the UA in CBCT scans can be performed manually, semiautomatically, and automatically. Manual measurements are considered the gold standard. However, they require a significant time investment, as the boundaries of the ROI must be manually marked in a 2D view for each layer of the scan. Fully automatic measurements, on the other hand, based on neural networks and machine learning algorithms, promise an accurate and rapid procedure. However, this method has thus far been described only experimentally^52^. The present study focused on the analysis of the UA as a routine diagnostic tool in a clinically feasible setting. For this purpose, only semiautomatic measurements are currently feasible.

Through algorithms for assessing the grayscale values of individual voxels, particularly by applying thresholding, semiautomatic programs are capable of accurately identifying the anatomical boundaries between soft tissue and air^53^. In 2010, El & Palomo investigated the precision and reliability of various commercially available semiautomatic programs for volume measurements of the UA (nasopharynx, oropharynx, and hypopharynx) via CBCT. Although they attributed high methodological reliability to all programs, comparisons with manual measurements revealed statistically significant differences for all programs^33^. A systematic review by Guijarro-Martínez & Swennen in 2011 reaffirmed the accuracy and reliability of three-dimensional (3D) analysis of the UA using CBCT^32^. However, Alsufyani et al. reported difficulties in making a definitive assessment of the current validity and reliability of CBCT-generated 3D models due to the limited number of studies in 2012^18^. In the same year, Weissheimer et al. examined the accuracy of six commercially available semiautomatic programs using an acrylic phantom and found that, among others, Dolphin3D exhibited an error of less than 2%^54^. Further studies by Schendel & Hatcher, Alsufyani et al., and Chen et al. demonstrated the high reliability and accuracy of various programs for airway measurement using CBCT^55–57^.

Although accurate data are undeniably crucial, the relevance of the mean differences and limits of agreement observed for each software in clinical and diagnostic contexts remains uncertain, given the absence of normative standards for airway volumes. Moreover, all the programs tested in this study demonstrated outstanding reliability. Thus, although volumetric data may exhibit variance, they maintain proportional equivalence. Consequently, based on our findings, semiautomatic software may serve as a viable tool for UA diagnosis, particularly in cases where normative values for the UA are lacking.

El & Palomo noted that the structure of the nasal passage, with its narrow twists through the choanae, poses a challenge for airway volumetrics in CBCT^33^. During the creation of a CBCT scan and the application of the Feldkamp algorithm, voxel grayscale averaging occurs, as described by Schendel & Hatcher^57^. Soft tissue structures are represented with low contrast. In narrow air-filled spaces between two soft tissue structures, such as the choanae, the systematic application of the algorithm results in voxel values that are disproportionately high.

In 2012, Alves et al. determined that a threshold of 73 was optimal for semiautomatic Dolphin measurements^58^. In the present study, this threshold was visually assessed in the axial and sagittal planes as well as based on the 3D model. The proposed threshold of 73 resulted in artifacts and significant deviations from the real anatomy of the oropharynx. Alves et al. examined the entire nasal passage and oropharynx in a single measurement, where the complex anatomy of the nasal passage may have required a higher threshold. An alternative explanation is provided by the study of Chen et al., which demonstrated that the use of different devices in combination with the same software leads to variable intra- and interrater values for the thresholds, resulting in partially significant differences in volumetrics^48^. Alves et al. utilized an iCAT device (Imaging Sciences International, Hatfield, PA) for image generation. The only study that confirmed Alves’ results used the same combination of an iCAT device and Dolphin software^59^. In the present study, a ProMax® 3D Mid + Pan (Planmeca Oy, Helsinki, Finland) was used.

At the time of the investigations, there were no studies available for Ro 5 and Ro 6 threshold values. The default settings of the programs yielded satisfactory results upon visual inspection. Planmeca does not provide detailed information on how the default values for “air” are determined. In October 2023, after the completion of this study, Fahham et al. published results on threshold values for Ro 6, indicating optimal values between 600 and 850^60^. However, these values were determined through a comparison with manual measurements using the ImageJ program and are therefore highly dependent on the evaluator^61^.

The accuracy and reliability of semiautomatic volume and cross-sectional measurements of the UA with Dolphin have been the subject of various studies, yielding partially conflicting results. In 2012, Weissheimer et al. examined the accuracy of Dolphin along with five other programs in measuring an acrylic phantom. Dolphin demonstrated a very low error rate of only 1%, indicating high precision^54^. Ghoneima and Kula et al. also validated Dolphin using an acrylic phantom and found no significant errors^62^. In contrast, in 2020, Torres et al. reported significant differences between Dolphin and InVivo in measuring three different airway phantoms. Dolphin generally overestimates the volume, while InVivo underestimates the volume^47^. One reason for the disparate results may lie in the different constructions and volume determinations of the phantoms. In another comparison by Lo Giudice in 2022, statistically significant differences were also observed between various programs and manual measurements. Dolphin performed the worst, with a mean error of 6.06 cm3 (78.26%)^63^.

In 2013, Souza et al. investigated the reliability of volumetry with Dolphin using 60 CBCT scans, yielding excellent ICCs ranging from 0.88 to 0.99^41^. A study by Mattos et al. in 2014 corroborated these findings^45^. The results of the present study even exceeded these findings (Table 4).

Studies on the application of the Romexis 5 criteria for UA examination can be found in the literature^64^. The software versions differ significantly in their approach to airway analysis: Romexis 5 utilizes the Regional Growth Tool (RGT), a versatile tool designed for measuring various cavities in the skull, including the upper airways. The algorithm underlying the RGT is not publicly disclosed. In some cases, when patients do not hold the tongue perfectly at the palate, portions of the oral cavity are erroneously included in the measurement, which limits precise volumetry.

Romexis 6, in contrast, introduces the specific Airway Extraction Tool (AET), which was developed exclusively for UA analysis. Compared to the RGT, it offers improved usability and a more streamlined workflow. The ROI is automatically defined, oriented along the ML, and not adjustable. While this specialization provides advantages in terms of ease of use, similar to Romexis 5, parts of the oral cavity can sometimes be erroneously included in the measurement, which may affect volumetric precision. The underlying algorithm of the AET, like its predecessor, remains proprietary information. The automation in ROI definition, while convenient, also means that users have less flexibility in adjusting measurement parameters.

ML presents a particular challenge. Airways vary among patients, exhibiting different geometries. An algorithm calculates the curvature when determining the ML, depending on the point offset. The cranial and caudal boundaries are automatically oriented along this curvature, preventing them from remaining parallel to the set image plane. While this problem can be addressed as described in the Materials & Methods section, it is impractical.

The MCA is determined with Dolphin and Romexis 5 parallel to the set horizontal image plane. Anatomically, the MCA, as feasible with Ro6, can be measured in relation to the axis of the airway. Aligning the image separately would significantly increase the time and complicate the measurements. Although significantly different results are expected, there are currently no studies investigating this issue.

## Limitations and future directions

Despite our comprehensive approach, several limitations of this study warrant consideration. The sample size of 29 patients, while sufficient for statistical analysis, may limit the generalizability of our findings to broader populations. The retrospective nature of our investigation introduces potential biases inherent to this study design. Our analysis was confined to three software programs (Dolphin, Romexis 5, and Romexis 6), which, although widely used, represent only a subset of available airway analysis tools. A notable limitation is the absence of validation against a gold standard, such as manual measurements or physical phantoms, which could have provided additional verification of our findings. Furthermore, all measurements were performed by a single rater; while this ensured consistency, it precluded the assessment of inter-rater reliability. The variable timeframe of postoperative imaging (3–12 months) may have introduced temporal bias in our results. Additionally, while we focused on software comparison, we did not extensively analyze the potential influence of patient-specific factors such as age, gender, or specific surgical procedures on the measurements. The proprietary nature of the software algorithms presents another limitation, as it restricts our ability to fully explain the observed differences between programs. Future studies should address these limitations through prospective design, larger sample sizes, multiple raters, and standardized imaging timeframes to enhance the robustness and generalizability of findings in this important field of research.

In light of these limitations, future research should focus on several key areas. Prospective studies with larger sample sizes and standardized imaging timeframes are needed to validate our findings. The inclusion of multiple raters and additional software programs would enhance the robustness of comparisons. Future investigations should also consider patient-specific factors such as age, gender, and specific surgical procedures in their analyses. Moreover, validation against a gold standard, such as manual measurements or physical phantoms, would provide valuable insights into the accuracy of these software tools. Lastly, collaborative efforts with software developers to understand the underlying algorithms could help explain the observed differences and potentially lead to more standardized measurement protocols across different programs.

## Clinical relevance

The analysis of the upper airway is essential for comprehending and managing obstructive sleep apnea syndrome (OSAS) and holds significant relevance in the domain of orthognathic surgery. Orthognathic surgery serves as a highly effective treatment modality for OSAS, with its efficacy frequently assessed through reductions in the Apnea–Hypopnea Index (AHI). Surgical interventions, such as maxillomandibular advancement (MMA), exert a profound influence on the anatomy of the upper airway, affecting both volumetric capacity and the minimal cross-sectional area (MCA). Notably, the MCA has been demonstrated to exhibit a negative correlation with the AHI, underscoring its potential utility in predicting AHI levels in patients with OSAS^65^.

Moreover, preoperative dimensions, gender, and the extent of mandibular advancement function as critical explanatory variables that, when analyzed collectively, can predict the 1-year postoperative volume and minimal area^66^. Existing literature has established a correlation between alterations in AHI and variations in airway volume, as well as a relationship between the degree of advancement during MMA and changes in volumetric measurements^35^. Conversely, mandibular setback procedures are associated with reductions in both volume and MCA, with the MCA being the most pertinent anatomical parameter linked to the pathogenesis of OSAS^67^.

Consequently, it is of paramount importance for surgeons to accurately evaluate the volume and MCA parameters of their patients. A variety of imaging modalities, including computed tomography (CT), magnetic resonance imaging (MRI), and cone-beam computed tomography (CBCT), are available for this purpose. Among these, CBCT offers distinct advantages, including lower costs compared to CT and MRI, reduced radiation exposure relative to CT, and fewer contraindications compared to MRI, thereby establishing it as the preferred imaging modality.

Numerous software programs have emerged to facilitate the measurement of volume and MCA. However, our study highlights that different software can yield variable interpretations of these measurements, which may directly influence therapeutic decisions and the assessment of treatment outcomes. This finding is particularly critical for surgeons who may utilize different software over time or refer to measurements obtained by colleagues employing varying programs.

## Conclusions

This study compared three software programs (Dolphin, Romexis 5, and Romexis 6) for evaluating upper airway changes following orthognathic surgery using CBCT. Our findings reveal:

1. High intra-rater reliability across all three programs, confirming their utility as reproducible measurement tools.
2. Significant differences in volumetric measurements between Dolphin and Romexis programs (*p* < 0.05), while no significant differences were found in minimal cross-sectional area (MCA) measurements (*p* = 0.069).
3. Variability in measurements across software platforms, highlighting the need for standardization in multi-center studies and meta-analyses.

These results have important implications:

1. Software choice can significantly impact airway volume assessments, potentially influencing clinical decisions in OSAS management and orthognathic surgery outcome evaluation.
2. The established correlation between airway parameters and clinical indicators (e.g., AHI) underscores the importance of accurate and consistent measurements.
3. Standardization of threshold values for airway segmentation across different CBCT device and software combinations is crucial for improving measurement consistency.

Our study demonstrates significant differences in the interpretation of oropharyngeal volume changes following orthognathic surgery between Dolphin and Romexis software programs. Despite standardized protocols, these discrepancies highlight the need for caution when comparing clinical data across different software platforms or institutions. While all three programs allow for reproducible and quantifiable measurements, the observed differences underscore the importance of consistency in software choice for longitudinal assessments. Despite these challenges, CBCT remains a valuable tool for upper airway assessment, offering advantages over other imaging modalities, particularly in the context of orthognathic surgery and OSAS management. In conclusion, while the analyzed software programs provide reliable tools for upper airway evaluation, the variability in their measurements necessitates careful interpretation and comparison of data. These findings have important implications for both clinical practice and research, emphasizing the need for standardization in CBCT-based airway analysis methodologies, especially when evaluating treatment outcomes in orthognathic surgery and managing patients with OSAS.

## Supplementary Information

Supplementary Information.

## Data Availability

https://mediatum.ub.tum.de/1740288. Login:reviewer-access-02 Pass: LwMx)!G*PrSm.&sLe$fAn.
