# Supplementary material for: Cone beam computed tomography based upper airway measurement after orthognathic surgery: a comparative evaluation of different imaging software
Source: Sci Rep. 2025 Feb 24;15:6638. doi: 10.1038/s41598-024-83890-7 (PMC11850604; doi:10.1038/s41598-024-83890-7)
Supplement: Supplementary file 1 — Supplementary Information. [file 41598_2024_83890_MOESM1_ESM.docx]

# Supplementary Information

## Bland Altman Analysis - Comparison of Software

### Volume

Fig. 2 Comparing Dolphin and Romexis 6 measuring V. Red/Midline=Mean, Green/Upper/Lower Line=LoA

Fig. 1 Comparing Dolphin and Romexis 5 measuring V. Red/Midline=Mean, Green/Upper/Lower Line=LoA

Fig. 3 Comparing Romexis 5 and Romexis 6 measuring V. Red/Midline=Mean, Green/Upper/Lower Line=LoA

### Minimal cross-sectional area

Fig. 5 Comparing Dolphin and Romexis 6 measuring MCA. Red/Midline=Mean, Green/Upper/Lower Line=LoA

Fig. 6 Comparing Romexis 5 and Romexis 6 measuring MCA. Red/Midline=Mean, Green/Upper/Lower Line=LoA

Fig. 4 Comparing Dolphin and Romexis 5 measuring MCA. Red/Midline=Mean, Green/Upper/Lower Line=LoA

## Bland Altman Analysis - Comparison of M1 and M2 for all Software packages and timepoints

### Volume

Fig. 12 Comparing V M1 and M2 for Romexis 6 at t0. Red/Midline=Mean, Green/Upper/Lower Line=LoA

Fig. 11 Comparing V M1 and M2 for Romexis 6 at t0. Red/Midline=Mean, Green/Upper/Lower Line=LoA

Fig. 9 Comparing V M1 and M2 for Romexis 5 at t0. Red/Midline=Mean, Green/Upper/Lower Line=LoA

Fig. 10 Comparing V M1 and M2 for Romexis 5 at t0. Red/Midline=Mean, Green/Upper/Lower Line=LoA

Fig. 7 Comparing V M1 and M2 for Dolphin at t0. Red/Midline=Mean, Green/Upper/Lower Line=LoA

Fig. 8 Comparing V M1 and M2 for Dolphin at t1. Red/Midline=Mean, Green/Upper/Lower Line=LoA

Table 7 Values for Figs. 7–12; all in cm^3^

|  | (V) Dolphin t0 Diff. M1/M2 | (V) Dolphin t1 Diff. M1/M2 | (V) Romexis5 t0 Diff. M1/M2 | (V) Romexis5 t1 Diff. M1/M2 | (V) Romexis6 t0 Diff. M1/M2 | (V) Romexis6 t1 Diff. M1/M2 |
| --- | --- | --- | --- | --- | --- | --- |
| Mean Difference | 0,145 | 0,260 | 0,317 | 0,245 | 0,122 | 0,017 |
| Standard deviation | 0,771 | 0,987 | 1,141 | 1,102 | 1,636 | 1,398 |
| LoA (upper limit) | 1,365 | 2,194 | 2,553 | 2,405 | 3,084 | 2,757 |
| LoA (lower limit) | -1,655 | -1,625 | -1,918 | -1,915 | -3,329 | -2,73 |

MCA

Fig. 14 Comparing MCA M1 and M2 for Dolphin at t1. Red/Midline=Mean, Green/Upper/Lower Line=LoA

Fig. 13 Comparing MCA M1 and M2 for Dolphin at t0. Red/Midline=Mean, Green/Upper/Lower Line=LoA

Fig. 15 Comparing MCA M1 and M2 for Romexis 5 at t0. Red/Midline=Mean, Green/Upper/Lower Line=LoA

Fig. 16 Comparing MCA M1 and M2 for Romexis 5 at t1. Red/Midline=Mean, Green/Upper/Lower Line=LoA

Fig. 18 Comparing MCA M1 and M2 for Romexis 6 at t1. Red/Midline=Mean, Green/Upper/Lower Line=LoA

Fig. 17 Comparing MCA M1 and M2 for Romexis 6 at t0. Red/Midline=Mean, Green/Upper/Lower Line=LoA

Table 8 Values for BAP Figs. 13–18; all in mm^2^

|  | MCA Dolphin t0 Diff. M1/M2 | MCA Dolphin t1 Diff. M1/M2 | MCA Romexis5 t0 Diff. M1/M2 | MCA Romexis5 t1 Diff. M1/M2 | MCA Romexis6 t0 Diff. M1/M2 | MCA Romexis6 t1 Diff. M1/M2 |
| --- | --- | --- | --- | --- | --- | --- |
| Mean Difference | 1,207 | -6,241 | 7,621 | 5,345 | 15,310 | -0,966 |
| Standard deviation | 10,213 | 49,023 | 20,201 | 13,836 | 26,444 | 38,320 |
| LoA (upper limit) | 21,225 | 89,845 | 47,216 | 32,464 | 36,520 | 74,142 |
| LoA (lower limit) | -18,811 | -102,32 | -31,974 | 21,775 | -67,140 | -76,073 |
